# E-PAtS trial: a protocol for a cluster randomised controlled trial of Early Positive Approaches to Support (E-PAtS) for families of young children with intellectual disabilities

**DOI:** 10.1136/bmjopen-2025-112783

**Published:** 2026-10-27

**Authors:** Rhys Williams-Thomas, Elinor Coulman, Jill Bradshaw, Debbie Austin, Cameron Bradshaw, Myrsini Gianatsi, David Gillespie, Gemma Grant, Richard P Hastings, Sungwook Kim, Richard May, Rachel McNamara, Peter Mulhall, Lydia Penrose, Stavros Petrou, Sophie Rose, Jeremy Segrott, Joanne Sweeney, Laurence Taggart, Serena Tomlinson, Vasiliki Totsika, Caitlin Williams, Nick Gore

**Affiliations:** 1 Centre for Trials Research, Cardiff University, Cardiff, UK; 2 Intellectual Disabilities Research Institute, University of Birmingham, Birmingham, UK; 3 The Challenging Behaviour Foundation, Chatham, UK; 4 Nuffield Department of Primary Care Health Sciences, University of Oxford, Oxford, UK; 5 University of South Wales, Pontypridd, UK; 6 Queen’s University Belfast, Belfast, UK; 7 DECIPHer Centre, Cardiff School of Social Sciences, Cardiff University, Cardiff, UK; 8 University of Kent, Canterbury, England, UK; 9 Division of Psychiatry, University College London, London, UK

**Keywords:** Randomized Controlled Trial, Family, MENTAL HEALTH, Caregivers

## Abstract

**Introduction:**

Approximately 2%–3% of children globally have intellectual disabilities, with around 300 000 in England. These children face cognitive, social and educational challenges, and their parents often experience high stress and reduced mental health. Early interventions are vital to support both child development and family well-being. The parent–child relationship in early years (ages 3–5) and parental well-being both play a key role in shaping later child outcomes. However, current interventions often focus on either child development or parental well-being, not both. There is a gap in group programmes tailored for families of young children with intellectual disabilities, especially those that include peer support and co-production. The Early Positive Approaches to Support (E-PAtS) programme was developed to address these gaps, showing promise in a feasibility trial.

**Methods and analysis:**

We are conducting a randomised controlled trial of the E-PAtS intervention. Trial design and delivery have been co-produced with family carers, following on from a successful feasibility trial. A recruitment period of 29 months will commence on 1 September 2023. After completing baseline assessments, families are randomised on a 1:1 basis either to the E-PAtS intervention or continue with usual care. Control group families will have access to the intervention following 12-month assessments. The intervention comprises an 8-week manualised group programme for the family carers of children with an intellectual disability. At 4 months and 12 months, participants are invited to complete repeat outcome assessments. The primary objective is to assess whether the E-PAtS intervention has a positive effect on parents/carers’ mental well-being at the 12-month time point, assessed by Warwick-Edinburgh Mental Well-Being Scale (WEMWBS). The primary treatment effect at 12 months will be estimated using a partially clustered linear mixed model of repeated WEMWBS measurements, adjusting for baseline score and site, with random effects for participants, families and intervention-arm E-PAtS groups. Secondary objectives include further exploration of mental health outcomes in both parent and child as well as health-related quality of life and an economic analysis.

**Ethics and dissemination:**

The Sponsors of this study are the University of Birmingham and University of Kent. This protocol received approval from Humanities and Social Sciences Ethics Committee, University of Birmingham (13/08/2025, Ref: ERN_3848-Jul2025), following initial approval from the Tizard Centre University of Kent Ethics Committee (16/08/2023, Ref: 0897) Study results will be disseminated through peer reviewed literature, academic conferences, ISRCTN registry, directly to participants and stakeholders and through dissemination events. Processes will be determined through partnership working with our Public and Patient Involvement and Engagement group.

**Trial registration number:**

ISRCTN11192967

**STRENGTHS AND LIMITATIONS OF THIS STUDY**

- The Early Positive Approaches to Support (E-PAtS) programme has been coproduced and is cofacilitated with parent/carers.
- The trial has benefited from Public and Patient Involvement and Engagement (PPIE) input at every stage by both having PPIE representation on management and steering meetings but also engaging with a separate PPIE group for discrete tasks such as retention strategies.
- The trial incorporates a health economics analysis and process evaluation.
- Some settings in which E-PAtS is delivered (ie, health services) are not in scope for this trial.
- Participants for this trial will not be blind to their allocation.

## Introduction

 Prevalence studies internationally suggest approximately 2%–3% of children and adolescents have intellectual disabilities.^1^ Based on Department for Education data published from the National Pupil Database, there are approximately 300 000 children of school age with intellectual disabilities in England.^2^ Young children with intellectual disabilities face, by definition, a number of cognitive, learning, social and everyday living challenges. These children also experience a range of other social, health and educational inequalities.^3^ Parents of children with intellectual disabilities often have elevated levels of stress, mental and physical health problems themselves. For example, research suggests that mothers of children with intellectual disabilities are approximately 1.5 times more likely than other mothers to experience depression,^4^ and similarly 1.4 times more likely than other mothers to have high levels of psychological symptoms indicative of mental health problems.

Given this research evidence, interventions and supports are needed that target both parental or family well-being and developmental outcomes for the child with intellectual disabilities. Developmental challenges for children with intellectual disabilities and inequalities affecting their families emerge early in life (certainly by ages 3 to 5 years) and so early intervention and support is an important individual family and larger policy priority.^5^ In addition to parental well-being, what parents do and the relationships they build with their child with intellectual disabilities have been shown to be crucial for the development of children with intellectual disabilities (as they are for all children). For example, the quality of the parent–child relationship at age 3 years has been shown to predict behaviour problems at age 5 years among children with intellectual disabilities.^6^ Negative dimensions of parenting in the early years (3 to 5 years of age) also mediated the effects of early maternal well-being on behaviour problems among children with intellectual disabilities at 7 and 11 years of age.^7^ Therefore, interventions that also target parenting practices/strategies and parent–child relationships could have significant potential to support families of young children with intellectual disabilities.

The existing evidence base for early family/parent-based interventions for families of children with intellectual disabilities is limited. There is some evidence relating to early intervention approaches that focus only on teaching new or extended skills to children with intellectual disabilities^8^ without any additional support for family members. Similarly, there are some intervention strategies from a psychological therapy perspective (such as cognitive behavioural therapy and more recently mindfulness-based approaches) that potentially reduce parental psychological distress in families of children with intellectual disabilities^9 10^ where the interventions do not also focus on child outcomes. Psychoeducation interventions may also be used in UK services, typically addressing one or more of three components^11^: information about disability, information about services and support available to families, and potentially a brief psychoeducational perspective on well-being.

Although such psychoeducation interventions have been evaluated with parents of preschool children,^12^ parents of adolescents approaching transition^13^ and with parents of adults,^14^ the evaluations are small and rarely use randomised controlled trial (RCT) designs.^11^ Other psychoeducation programmes have focused only on services and improving access to services,^15^ including by increasing parents’ confidence to access services.^16^ Parenting interventions, which focus on both child and parental outcomes, were reviewed systematically by National Institute for Health and Care Excellence (NICE) to inform the ‘Mental Health Problems in People with Learning Disabilities’ clinical guideline.^17^ The parenting programmes reviewed by NICE also did not explicitly target parent well-being but focused on problems related to the child (eg, behaviour problems). In terms of evidence gaps, NICE found no evidence relating to group parenting programmes designed specifically for parents of young children with intellectual disabilities, without a specific focus on a problem related to the child, and with the explicit aim of improving parent psychosocial well-being. Therefore, there is a gap in availability of and evidence for, suitable group parenting programmes.

A further limitation is the lack of approaches coproduced with families^18^ or including family members themselves as partners in supporting other families who have a child with intellectual disabilities. This is despite evidence that parents’ lived experience can be beneficial in supporting families of disabled children more generally. For example, a recent systematic qualitative synthesis of research in which parents offer peer support to other parents of children with disabilities^19^ identified four benefits of peer-to-peer support: shared social contact with other parents, learning from the experiences of other parents, parent supporters gaining self-confidence and expertise, and all participants valuing the opportunity to support others in group contexts.

In terms of usual practice (UP), the majority (70%) of family carers of children with intellectual and developmental disabilities aged 0–6 in the UK reported having received no support for either themselves or their families in the last 12 months when surveyed.^20^ More than 75% of family carers who responded indicated that they had an unmet need for early support.^21^ Access to early support was linked to a child having a diagnosis and carer education level whereas unmet need for early support was linked with levels of economic deprivation.^22^

The Early Positive Approaches to Support (E-PAtS) group programme was designed for and with families of children with intellectual disabilities to improve psychosocial outcomes for family carers and developmental outcomes for children, and to directly address gaps in research and services. A feasibility trial of E-PAtS^23^ satisfied progression criteria, found E-PAtS was received favourably by family carers and was associated with improvements in family carer well-being. The current study will determine whether E-PAtS can improve health and social outcomes for family carers and their children with intellectual disabilities.

## Methods and analysis

### Objectives/Aim

#### Primary objectives

To assess whether E-PAtS plus UP is effective in leading to more positive mental well-being compared with UP alone for family carers of young children with intellectual disabilities at 12 months postrandomisation.

#### Secondary objectives

To examine the impact of E-PAtS plus UP compared with UP alone on: (1) family carer mental health (depression, anxiety); (2) family carer perceptions of the child with intellectual disabilities and confidence in parenting; (3) behavioural and emotional problems in the child; (4) levels of child pro-social behaviour and adaptive skills (including sleep); (5) child health-related quality of life; (6) other family impacts (eg, family functioning); (7) use and cost of health and social care services and broader services and (8) cost-effectiveness.

#### Process evaluation objectives

To examine the experiences of E-PAtS facilitators, participants and non-participating family carers in the intervention arm and second family carers who did not attend E-PAtS. To examine variation including with regards, adherence, intervention training, delivery and fidelity, intervention receipt mechanisms, provision and utilisation of services, future implementation and UP.

### Primary outcomes measure

The primary outcome measure will be the Warwick-Edinburgh Mental Well-Being Scale (WEMWBS) to measure parental/carers’ mental well-being^24^ at 12 months post-randomisation.

### Secondary outcomes measure(s)

Secondary outcome measures include: (1) perceptions of parenting: Me as a Parent Scale short form^25^; (2) parental/carer anxiety and depression: Hospital Anxiety and Depression Scale^26^; (3) child with intellectual disability adaptive development: The GO4KIDDS Brief Adaptive Scale^27^ adapted to include an additional communication item (completed by parents/carers of children 18 months and above); (4) child emotional and behavioural development: The Strengths and Difficulties Questionnaire (completed by parents/carers of children 2 years and above)^28^; (5) child sleep: Single Sleep question from Millennium Cohort Survey^29^; (6) child health-related quality of life: The Paediatric Quality of Life Inventory Version 4.0 Generic Core Scales (completed by parents/carers of children 2 years and above); (7) family functioning: Family APGAR Scale^30^; (8) support for family: Family Support Scale^31^; (9) parent health-related quality of life (EuroQol 5-Dimension 5-Level questionnaire (EQ-5D-5L)^32^) and (10) Client Service Receipt Inventory (CSRI) modified from the feasibility study.^23^

### Process evaluation outcome measure(s)

Qualitative interviews following 12-month data collection with: intervention facilitators and provider representatives; participants in the intervention arm (including those who do not complete the intervention); second parents/carers who did not attend E-PAtS groups personally but where another parent/carer in the family did.

In addition, the following quantitative data will be recorded by facilitators at intervention sessions as part of the process evaluation: facilitator self-completed checklists for each intervention session; attendance of intervention at a family level (attendance of 5/8 sessions by 1 or more family members).

### Trial design

The trial is a two-arm cluster randomised wait-list control trial with embedded process and economic evaluations of E-PAtS in addition to UP compared with UP alone. The primary outcome is family carer well-being (as measured by the WEMWBS) at 12 months post-randomisation. The study end date is defined as the date the last participant completes follow-up.

### Study setting and site selection

Participants will be recruited from England, Scotland, Wales and Northern Ireland across four research centres. Third sector and local authority funded service provider organisations will be selected to take part in the trial if they are able to deliver E-PAtS with a minimum group number of 6 families and are willing to refer potentially eligible families to the research team.

### Public and patient involvement and engagement

E-PAtS is a coproduced intervention (Intervention) and family carers have contributed towards the design and delivery of this trial. The trial team includes a family carer co-applicant and family carer representative co-applicant. In addition, the trial will have a Public and Patient Involvement and Engagement (PPIE) advisory group comprised of parent/carers with lived experience of raising a child with intellectual disabilities. The role of this group will be for members to use their lived experience to help inform research activities, including reviewing and co-creating recruitment materials and helping to write information to share the outcomes of the study. Family carer representatives will also be members of governing groups for this study (ie, the Trial Steering Committee and independent Data Management Ethics Monitoring Committee).

### Participant selection

Participants for this study will be adult family carers of children with intellectual disabilities. At least one family carer (first family carer) is recruited from each family, with a second family carer then recruited where possible. First family carers attend the E-PAtS intervention if randomised to do so. Second family carers are able to attend all, some or none of the E-PAtS sessions.

### Inclusion criteria

#### First family carer

First family parents/carers will be eligible if they (1) are available to attend the E-PAtS intervention; (2) are 18 years old or older; (3) have sufficient verbal English language enabling completion of outcome measures; (4) can provide informed consent and (5) have at least one child with an intellectual disability aged 0 months to <7 years (previously 18 months to <6) who lives the majority of the time with the identified family carer. The identified child with intellectual disabilities must meet the following criteria: have a diagnosis or be in the process of exploring a diagnosis or presentation related to intellectual and/or developmental disability and has a standard composite score on the Vineland-3 Comprehensive Level Adaptive Behaviour Scale (children aged <3 years) or on the Domain-level version of the Vineland Adaptive Behaviour Scales III (children aged 3 years and above) of <80 when screened.^33^

#### Second family carer

First family carers/parents will be eligible if they: (1) are ≥18 years old; (2) have sufficient verbal English language enabling completion of outcome measures; (3) have at least weekly contact with the identified child with intellectual disabilities and (4) can provide informed consent.

### Exclusion criteria (first and second family carers)

First and second parents/carers will not be eligible if (1) they are enrolled in another group parenting programme at the point of screening; (2) they are enrolled in another programme of personal psychological therapeutic support at the point of screening; (3) they have previously participated in an E-PAtS group; (4) their child with intellectual disabilities is in a 24-hour residential placement or is in a foster placement due to end before the 12-month postrandomisation data point; (5) there are current child protection concerns relating to the identified child with intellectual disability and (6) the family is recognised to be in a state of current crisis and unable to cope/a score of 8–10 on the 10-point Brief Family Distress Scale where screened.^34^

### Sample size calculation

A total of 458 families (229 per arm) will be randomised in this study, to include a total of approximately 594 family carers (297 per arm). The sample size is estimated for 90% power, with a two-sided type I error rate of 5%. The established minimally important difference on the selected primary outcome measure, the WEMWBS scale, is 3 points,^24 35^ assuming an SD of 11 points (ie, a standardised effect size of 0.27) and a correlation of 0.5 between family carers’ baseline and follow-up WEMWBS scores. This is lower than the correlation observed in the feasibility study (0.57) and is in line with baseline to follow-up correlations reported elsewhere.^36 37^ Family carer scores are expected to cluster within families (intraclass correlation of 0.4 assuming an average of 1.3 participants per family), but families are not expected to cluster within intervention groups. These assumptions were informed by the results of our feasibility trial,^38 39^ in which the intraclass correlation of responses within families was less than 0.3, with no evidence of clustering by intervention group. A 20% loss to follow-up is allowed, which is lower than we found in our feasibility study but is expected given the enhanced retention strategy to be used. At the end of the internal pilot, the assumptions that were assessed without knowledge of outcome data or treatment allocation were loss to follow-up, cluster sizes (participants per family and families per group) and variability of cluster sizes. The sample size was revised, based on updated information available during the trial after 136 out of 458 families were randomised; 16 months after the recruitment had started. The trial will randomise 186 families (93 per arm), corresponding to approximately 240 family carers (120 per arm). The revised sample size provides 80% power to detect the established minimally important difference of 3 points on the WEMWBS, using a two-sided 5% significance level. The revised assumptions were an SD of 7.7, a correlation of 0.31 between baseline and 12-month WEMWBS scores, an average of 1.3 family carers per family, an intraclass correlation coefficient of 0.44 for parent/caregivers within families, a coefficient of variation in family size of 0.34 and 89% retention.

### Intervention

E-PAtS is a manualised programme for groups of 8–12 families of children with intellectual disabilities, with 1–2 adults per family attending. E-PAtS is facilitated by a trained family carer and practitioner dyad working in partnership. E-PAtS aims to support family carer well-being, proactive access to local support systems and positive development for young children with intellectual disabilities. The E-PAtS programme comprises: (1) an individual preparation interview for family carers with facilitators/a representative from their organisation, (2) 8 (typically weekly), 2.5-hour group sessions and (3) a personalised workbook and associated resources. Full details of the co-production of E-PAtS, associated logic model and content are provided elsewhere.^35^

In the current trial, E-PAtS will be provided in addition to UP. E-PAtS will be delivered by practitioner and family carer facilitators who have completed manualised E-PAtS training and are working in third sector or local authority settings across the UK.

### UP/comparator

The comparator intervention for this study will be UP. UP includes any service (mainstream and specialised) provided to families and their children with intellectual disabilities that may vary by function (eg, parent support, intervention for the child), and/or by the main recipient (eg, the parent, the child with intellectual disabilities, the whole family). UP may include parenting support or psychological therapy for psychosocial health, but we will not recruit families already receiving a recognisable parenting programme intervention or psychological therapy for mental health problems at the time of baseline assessments (see Exclusion criteria). UP will be recorded in the study in interviews with family carers and through service use data. Following completion of their 12-month data collection, UP participants will be offered E-PAtS in line with the waitlist control design.

### Participant identification

Participants will be identified and referred to the research team by service providers (with appropriate support by researchers) in local areas using a flexible multipoint recruitment method. This method includes family carers in contact with charitable support organisations, local authority services, special schools and nurseries, after-school and weekend services for children with special educational needs and disabilities, parent/family support groups, social and local media advertising and researcher-led information sessions. Family carers can also self-refer by contacting service providers directly or the research team.

### Recruitment and data collection methods

A Participant Information Sheet (PIS), Screening Eligibility and Main Consent Forms (online supplemental file 1) will be sent to first family carers by researchers, with sufficient time (ie, 24 hours) given to review information. A screening/eligibility interview will then be conducted over the ‘phone or by video conferencing by researchers. Screening measures will be taken with verbal consent and contact details and preferences for future outcome measure completion recorded electronically. Where ineligibility status may change in the future, contact details will be stored with consent for future contact, and eligibility checks repeated at a future point.

The First Family Carer will complete informed consent, either online via an email link to the online consent or verbally with a researcher over the telephone or on video conferencing, as per participant preference. Baseline data will be collected in the same way, as per participant preference. Where the First Family Carer identifies a Second Family Carer, the Second Family Carer will also undergo eligibility screening, consent procedures, and baseline data collection following the same processes.

Once consent and baseline measures are collected for all participants in each family (and a sufficient number of families have been identified, see ‘Randomisation’) the family will be randomised to intervention or control. Participants will be informed of their study allocation by the research team via telephone or videoconferencing. The service delivery team, informed of the family’s participation, will provide the family with details of attending the E-PAtS intervention, if applicable.

Participant questionnaire data for this study are collected at baseline, 4 months postrandomisation and 12 months postrandomisation, collected either remotely with the support of a researcher or via online link as per participant preference. See table 1 for details of all outcome measures and data collection time points.

**Table 1:** Schedule of participant recruitment, interventions and assessments

Procedures		Study time points				
	Screening	Baseline	Randomisation	Intervention period	4-month follow-up	12-month follow-up
Consent for eligibility measures	X					
Eligibility	X					
Vineland-3 Comprehensive Level Adaptive Behaviour Scale (children aged 0 months to <3 years) or Vineland Adaptive Behaviour Scales III Domain Version (children 3 years and above)	X					
Brief family Distress Scale	X					
Informed consent		X				
Contacts form	X					
Demographics		X				
Randomisation			X			
Intervention				X		
Warwick-Edinburgh Mental Well-Being Scale—parental/carer psychological well-being		X			X	X
Me as a Parent Scale short form		X			X	X
Parental anxiety and depression—Hospital Anxiety and Depression scale		X			X	X
Adaptive development: The GO4KIDDS Brief Adaptive Scale adapted to include an additional communication item (completed by parents/carers of children 18 months and above)		X			X	X
Single Sleep question from Millennium Cohort Survey		X			X	X
The Strengths and Difficulties Questionnaire (completed by parents/carers of children 2 years and above		X			X	X
Child health-related quality of life: measured using the Paediatric Quality of Life InventoryTM Version 4.0 Generic Core Scales (completed by parents/carers of children 2 years and above)		X			X	X
Family functioning: Family APGAR scale		X			X	X
Support for family: Family Support Scale		X			X	X
Parent health-related quality of life (EuroQol 5-Dimension 5-Level questionnaire (EQ-5D-5L))		X			X	X
Client Service Receipt Inventory modified		X			X	X
Demographics form (facilitators)				X		
Session checklists (facilitators)				X		
Session Attendance logs (facilitators)				X		
Participant qualitative interviews						X
Qualitative interviews with facilitators and provider representatives						X
E-PAtS RCT weekly activity log record				X		

E-PAtS, Early Positive Approaches to Support; RCT, randomised controlled trial.

### Assessments

The schedule of participant recruitment, interventions and assessments is detailed in table 1.

### Retention strategy

The following strategies will be used to maximise retention: (1) Incentivising completion of all outcome data (First and Second Family Carers will each receive a £15 voucher after completion of baseline and 4-month data and a £30 voucher following completion at the 12 months post-randomisation data point, with those participants taking part in interviews as part of the process evaluation receiving a further £20 voucher). (2) Collecting multiple participant contact details, with consent, to support communications. (3) Following recommendations from our PPIE partners and feedback from participants in our feasibility study, enhancing communication with participants throughout the trial, including via a coproduced retention video, newsletters and work with local partners supporting people for whom English is not a first language. (4) Using a minimum dataset (comprising demographics and the primary outcome measure only at baseline and primary measure only at follow-up) if (after follow-up requests from researchers) participants have been unable to complete the full dataset.

### Randomisation

The units of randomisation are families including one or two family carer participants. Cluster-randomisation was chosen to avoid contamination between family members and to reflect intended implementation at the family level. Following consent, families will be randomised 1:1 to E-PAtS and UP or UP and waitlist control, stratified by site. Families will be randomised in batches of 6 to 16 two-weeks before the planned E-PAtS programme starts at each service provider, using randomly permuted blocks of varying size to reduce imbalance and maintain allocation concealment.

### Blinding

Participants and intervention facilitators will not be blind to allocation. However, the statistician carrying out the main statistical analyses will remain blind to allocation until the point the analysis is completed. Researchers collecting participant data remotely will use a standardised data collection script to minimise the risk of participants revealing allocation. If a researcher is made aware of the allocation of a participant, this will be recorded, and a different researcher will then be required to support any data collection for the participant going forward.

### Data management and security

Source data will be online or paper versions of the case report forms (CRFs)/questionnaires. All data will have unique identifiers. Hard copy CRFs/questionnaires will contain a unique identifier (participant Identification, PID), initials, and partial date of birth. Online CRFs/questionnaires (using a secure database) will be accessible via an online link and PID, initials and partial date of birth. Researchers will enter hardcopy data into the secure database, accessible only to appropriately trained personnel via username and password. The database will be housed on local servers managed by Cardiff University staff in accordance with legislation. Data validation will be performed at entry to reduce missing or unexpected data. All changes will be recorded in the audit log. Identifiable information will be encrypted and stored separately from research data.

Qualitative interviews will be audio recorded and later transcribed to support analysis. Recordings will be securely stored on password-protected computers at the research centres or OneDrive cloud with two-factor authentication. Recordings will be transcribed at site and pseudonymised transcripts checked against the recording before the recording is deleted. A separate recording of the participant giving verbal consent will be maintained (if applicable). Pseudonymised transcripts will be securely transferred to the study team at the Centre for Trials Research (CTR) by Fastfile. All files will be encrypted. A data management plan will be developed to outline the details of how data will be collected, transferred, stored and accessed by the team.

The CTR will preserve participant confidentiality and only disclose or reproduce identifiable information with specific consent. Data will be stored securely and registered with the General Data Protection Regulation 2016.

### Analysis

#### Internal pilot analysis

An internal pilot with predefined progression criteria will be included, assessed at the 4-month point for 25% of families, planned between months 6 and 14 of the study (9 months in total; incorporating the first 5 months of recruitment and 4-month follow-up data collected for families recruited during this period). Internal pilot progression criteria are set out in table 2. Rates are given overall, based on an average recruitment rate of 27 families per month.

**Table 2:** Internal pilot progression criteria

Progression criteria	Red (full trial not feasible)	Amber (proceed with optimisation)	Green (proceed without modification)
Recruitment (total and proportion of approached)			
No. of families recruited	<60% (67 or fewer)	60%–84% (68–96 families)	≥85%–100% 97–114 families
No. primary carers providing consent/approached	<40%	40%–49%	≥50%
Retention (4-month follow-up time point)			
	Less than 50%	50%–79%	≥80%–100%
Adherence			
	Less than 60%	60%–74%	≥75%–100%

#### Main statistical analysis

Baseline demographic characteristics will be summarised descriptively. Where appropriate, key demographic characteristics of the recruited sample, such as ethnicity, will also be descriptively compared with relevant UK population data. These comparisons will not be used to formally assess the representativeness of the trial sample. Data will be summarised using frequencies with percentages, means with SD, and medians with IQRs as appropriate. Differences in WEMWBS^24 35^ scores will be estimated at follow-up using a partially clustered linear mixed model to account for clustering of participants within families and intervention groups. Model assumptions will be assessed using diagnostic plots. Normality will be assessed using quantile–quantile plots of the model residuals, while residual-versus-fitted plots will be examined to assess linearity and homoscedasticity. The analysis population will be defined using the intention-to-treat (ITT) approach. WEMWBS scores will be treated as continuous, and baseline WEMWBS score will be included as a covariate in all analyses. The estimated intervention effect will be reported with a 95% CI and the intervention considered successful if zero is not within the 95% CI for the mean difference in WEMWBS at follow-up. The trial is powered to detect a clinically meaningful difference between intervention and control groups, using an established minimal important difference of 3 points on the WEMWBS scale. Missing outcome data will be summarised by trial arm and time point, including recorded reasons for missingness where available. The partially clustered linear mixed model will use all available outcome data and provide estimates under a missing-at-random (MAR) assumption.

Secondary outcomes will be analysed similarly. We will correct for multiple hypothesis testing by controlling the false discovery rate.^40^ The study is not powered to reliably detect differences in the secondary outcomes. In the analysis, the effect of being offered E-PAtS (ITT) will be investigated. To also investigate the effect of E-PAtS receipt, a complier average causal effect (CACE) analysis will be used.^41^ Families who have attended at least five of the eight sessions in the E-PAtS programme will be considered compliers. CACE with other definitions of compliance will be explored, such as intervention initiation (attendance of at least one session) or the number/pattern of sessions attended. For child outcomes, an additional sensitivity analysis will be conducted in which only the first carers’ responses will be considered. The potential for differential effects of the E-PAtS programme on WEMWBS scores by intended mode of delivery (online vs in-person) will be explored.

### Process evaluation

A mixed methods process evaluation will describe implementation, refine the E-PAtS logic model through examining intervention mechanisms, and consider the role of context in shaping intervention implementation and mechanisms. Consent to take part in the process evaluation will be collected separately from the main trial.

Qualitative interviews with facilitators and participants will explore implementation processes, intervention mechanisms—including receipt of activities by participants and group processes, the role of contextual factors, and interrogate patterns in the quantitative data. We will conduct interviews subsequent to the 12-month data collection.

Quantitative methods will be used to assess recruitment rates/patterns from baseline data and intervention fidelity across service delivery sites and will be reported descriptively. Qualitative data will be analysed using a reflexive thematic analysis approach, informed by, but not limited to, the broad categories as described in MRC guidance^42^ as a framework to describe implementation processes, examine intervention mechanisms and context.

### Economic evaluation

A within-trial economic evaluation, conducted from the public sector perspective, will estimate the cost of E-PAtS, including: (1) staff costs (eg, supervision, planning and delivery); (2) travel costs by programme facilitators and (3) other costs associated with delivering E-PAtS (eg, online platform costs (where applicable), reimbursed childcare costs). Broader resource utilisation will be captured through a modified CSRI questionnaires developed within our feasibility trial and completed at baseline and at 4 months and 12 months postrandomisation. Unit costs for resource inputs will largely be based on the unit costs compendium compiled as part of the feasibility trial.^23 39^

Responses to EQ-5D-5L^32^ health-related quality of life questionnaires will be converted into health utilities using the utility algorithm recommended by NICE at the time of analysis for the purposes of quality-adjusted life-year (QALY) estimation. QALY profiles for each first family parent/carer will be estimated as the area under the baseline-adjusted utility curve across the baseline, 4 and 12 months postrandomisation data using the trapezoidal rule.^43^ A cost–utility analysis will be conducted and expressed in terms of incremental cost per QALY gained. Bivariate regression of costs and QALYs, with multiple imputation of missing data, will be conducted to generate within-trial estimates of incremental cost-effectiveness associated with E-PAtS. Sensitivity analyses will assess the impact of areas of uncertainty surrounding aspects of the economic evaluation. Cost-effectiveness acceptability curves will be used to show the probability of cost-effectiveness of E-PAtS at alternative threshold values for an additional QALY held by decision-makers.^44^ A separate cost–consequences analysis will be conducted that presents the disparate costs and consequences associated with E-PAtS in disaggregated form. Before analysing the trial data, we will check for missing values and determine their causes using Faria *et al*’s framework.^45^ We will analyse the nature of this missing data to see if it qualifies as MAR. If it is MAR, we will apply multiple imputation methods.

### Adverse event reporting

There are no expected adverse events related to the intervention or research procedures. The Ethics Committee have not requested that adverse events should be reported for this study.

However, should any member of the research team become concerned at any point about the well-being or safety of a participant or their child, study staff will follow a study-specific Standard Operating Procedure for dealing with harm which will be explained to participants during the consent process and highlighted explicitly in PISs.

### Ethics and dissemination

#### Study governance

The sponsors of this study are the University of Birmingham and University of Kent. This protocol received approval from the Humanities and Social Sciences Ethics Committee, University of Birmingham (13/08/2025, Ref: ERN_3848-Jul2025), following initial approval from the Tizard Centre University of Kent Ethics Committee (16/08/2023, Ref: 0897)

A collaboration agreement including a data sharing agreement has been signed by all relevant parties and a site agreement will be completed and signed with each provider delivery site. A Steering Committee will provide independent oversight to the Trial Management team and an independent Data Monitoring and Ethics Committee will monitor the trial and report to the Steering Committee.

#### Auditing

No independent audits are planned.

#### Confidentiality

All data will be kept for 15 years in line with the Sponsor’s Research Governance Framework Regulations for clinical research. Electronic data will be stored confidentially on password protected servers maintained on University networks. All hard copy forms will be stored in locked filing cabinets. For the process evaluation, all interviews will be video/audio recorded and later transcribed to support analysis. Recordings will be securely stored on password protected computers at the research centres or OneDrive cloud with two-factor authentication. Audio files will be transcribed and pseudonymised. No identifiable data will be published.

#### Dissemination policy

Outputs from the E-PAtS RCT will include open access peer reviewed journal articles in international academic journals, at national and international academic conferences and at University public engagement events. All publications and presentations relating to the trial will be supported by PPIE processes and authorised by the Trial Management Group.

#### Study status

This manuscript has been drafted according to version 10.0, 30 July 2025. The recruitment start date and the approximate recruitment end date are 1 September 2023 and 31 January 2026, respectively. The protocol has been written according to the Standard Protocol Items: Recommendations for Interventional Trials (SPIRIT) statement, the intervention according to the template for intervention description and replication (TIDieR) checklist, and the final report will follow the Consolidated Standards of Reporting Trials (CONSORT) statement. The trial is registered on the ISRCTN registry id: 11192967.

#### Changes to the protocol

Two substantive changes have been made to the protocol since trial opening. To maximise participant recruitment, we have sought to optimise the pool of potentially eligible families and mirror delivery of E-PAtS in real-world settings. First, a change has been made to inclusion criteria for participants, extending the age range of children with intellectual disability to aged 0 months to <7 years (where this was previously 18 months to <6 years). Second, a change was made to delivery format to allow E-PAtS groups to be delivered online (using pre-existing intervention manuals for online delivery) or face-to-face.

## Discussion

Children with intellectual disabilities and their families have high support needs but support in the early years is often very limited or non-existent. E-PAtS is a support programme for family carers of young children with intellectual disabilities that focuses on carer well-being, access to services and children’s positive development. A prior feasibility study of E-PAtS^36^ demonstrated that it was possible to recruit families within an RCT design and that this was acceptable to families. The feasibility study also highlighted the importance of attending to recruitment challenges. The current RCT of E-PAtS has the following notable strengths:

1. Grounded in long-term and substantive PPIE input: The E-PAtS intervention has been coproduced with family carers and is cofacilitated by family carers and partners working in partnership; PPIE coapplicants and partners supported development of the study design and strategies for recruitment and retention and a PPIE group will advise on processes, materials and dissemination activities throughout the study.
2. Study design (a two-arm cluster randomised wait-list control trial, with multipoint recruitment across the UK) is a robust design that builds on findings from prior feasibility testing and analysis employs appropriate modelling methods that suitably adjust for clustering and include carefully considered sensitivity analysis.
3. Inclusion of embedded process evaluation and health economics analysis.

The study has the following limitations:

1. Health services are not in scope for this study. This was a pragmatic decision (based on available resources and timelines) to focus on a single setting, that is, the most common setting for delivery in practice. We have no reason to suggest the intervention itself would work differently in health settings.
2. It was not possible to blind participants to allocation due to the nature of the intervention. However, outcome assessors will be blind to allocation.

## Supplementary material

10.1136/bmjopen-2025-112783 online supplemental file 1

## References

[1] Maulik PK, Mascarenhas MN, Mathers CD (2011). Prevalence of intellectual disability: a meta-analysis of population-based studies. Res Dev Disabil.

[2] England PH (2020). People with learning disabilities in England: chapter 1, education and children’s social care.

[3] Emerson E, Hatton C (2014). Health inequalities and people with intellectual disabilities.

[4] Singer GHS (2006). Meta-analysis of comparative studies of depression in mothers of children with and without developmental disabilities. Am J Ment Retard.

[5] Schuengel C, van Rest MM, Stanford CE (2019). Impact of Research About the Early Development of Children With Intellectual Disability: A Science Mapping Analysis. Front Educ.

[6] Totsika V, Hastings RP, Vagenas D (2014). Parenting and the behavior problems of young children with an intellectual disability: concurrent and longitudinal relationships in a population-based study. Am J Intellect Dev Disabil.

[7] Totsika V, Hastings RP, Emerson E (2020). Early Years Parenting Mediates Early Adversity Effects on Problem Behaviors in Intellectual Disability. Child Dev.

[8] Eldevik S, Jahr E, Eikeseth S (2010). Cognitive and adaptive behavior outcomes of behavioral intervention for young children with intellectual disability. Behav Modif.

[9] Dykens EM, Fisher MH, Taylor JL (2014). Reducing distress in mothers of children with autism and other disabilities: a randomized trial. Pediatrics.

[10] Singer GHS, Ethridge BL, Aldana SI (2007). Primary and secondary effects of parenting and stress management interventions for parents of children with developmental disabilities: a meta-analysis. Ment Retard Dev Disabil Res Rev.

[11] Glidden LM, Floyd FJ, Hastings RP, Glidden LM, Abbeduto L, McIntyre LL (2021). APA handbook of intellectual and developmental disabilities.

[12] Hsieh RL, Hsieh WH, Lee WC (2016). Short-term family-centered workshop for children with developmental delays enhances family functioning and satisfaction: A prospective clinical trial. Medicine (Baltimore).

[13] DaWalt LS, Greenberg JS, Mailick MR (2018). Transitioning Together: A Multi-family Group Psychoeducation Program for Adolescents with ASD and Their Parents. J Autism Dev Disord.

[14] Lunsky Y, P Hastings R, Weiss JA (2017). Comparative Effects of Mindfulness and Support and Information Group Interventions for Parents of Adults with Autism Spectrum Disorder and Other Developmental Disabilities. J Autism Dev Disord.

[15] Taylor JL, Hodapp RM, Burke MM (2017). Training Parents of Youth with Autism Spectrum Disorder to Advocate for Adult Disability Services: Results from a Pilot Randomized Controlled Trial. J Autism Dev Disord.

[16] Picard I, Morin D, De Mondehare L (2014). Psychoeducational Program for Parents of Adolescents With Intellectual Disabilities. Policy Practice Intel Disabi.

[17] National Institute for Health and Care Excellence (2016). Mental health problems in people with learning disabilities: prevention, assessment and management.

[18] Bovaird T (2007). Beyond Engagement and Participation: User and Community Coproduction of Public Services. Public Adm Rev.

[19] Shilling V, Morris C, Thompson-Coon J (2013). Peer support for parents of children with chronic disabling conditions: a systematic review of quantitative and qualitative studies. Dev Med Child Neurol.

[20] Sapiets SJ, Totsika V, Hastings RP (2021). Factors influencing access to early intervention for families of children with developmental disabilities: A narrative review. J Appl Res Intellect Disabil.

[21] Sapiets SJ, Hastings RP, Stanford C (2023). Families’ Access to Early Intervention and Supports for Children With Developmental Disabilities. J Early Interv.

[22] Sapiets SJ, Hastings RP, Totsika V (2024). Predictors of Access to Early Support in Families of Children with Suspected or Diagnosed Developmental Disabilities in the United Kingdom. J Autism Dev Disord.

[23] Coulman E, Gore N, Moody G (2022). Early positive approaches to support for families of young children with intellectual disability: the E-PAtS feasibility RCT. *Public Health Res*.

[24] Tennant R, Hiller L, Fishwick R (2007). The Warwick-Edinburgh Mental Well-being Scale (WEMWBS): development and UK validation. Health Qual Life Outcomes.

[25] Matthews J, Millward C, Hayes L (2022). Development and Validation of a Short-Form Parenting Self-Efficacy Scale: Me as a Parent Scale (MaaPs-SF). J Child Fam Stud.

[26] Snaith RP (2003). The Hospital Anxiety And Depression Scale. Health Qual Life Outcomes.

[27] Perry A, Taheri A, Ting V (2015). The GO4KIDDS Brief Adaptive Scale. J Appl Res Intellect Disabil.

[28] D’Souza S, Waldie KE, Peterson ER (2017). Psychometric Properties and Normative Data for the Preschool Strengths and Difficulties Questionnaire in Two-Year-Old Children. J Abnorm Child Psychol.

[29] National Centre for Social Research (2008). Millennium cohort study sweep 3 questionnaire documentation.

[30] Smilkstein G (1978). The family APGAR: a proposal for a family function test and its use by physicians. J Fam Pract.

[31] Dunst CJ, Jenkins V, Trivette CM (1984). The family support scale: reliability and validity. Journal of Individual, Family, and Community Wellness.

[32] van Hout B, Janssen MF, Feng Y-S (2012). Interim scoring for the EQ-5D-5L: mapping the EQ-5D-5L to EQ-5D-3L value sets. Value Health.

[33] Sparrow SS, Saulnier CA, Cicchetti DV (2016). Vineland-3: Vineland Adaptive Behavior Scales. Manual.

[34] Weiss JA, Lunsky Y (2011). The Brief Family Distress Scale: A Measure of Crisis in Caregivers of Individuals with Autism Spectrum Disorders. J Child Fam Stud.

[35] Maheswaran H, Weich S, Powell J (2012). Evaluating the responsiveness of the Warwick Edinburgh Mental Well-Being Scale (WEMWBS): group and individual level analysis. Health Qual Life Outcomes.

[36] Borm GF, Fransen J, Lemmens WAJG (2007). A simple sample size formula for analysis of covariance in randomized clinical trials. J Clin Epidemiol.

[37] Walters SJ, Jacques RM, Dos Anjos Henriques-Cadby IB (2019). Sample size estimation for randomised controlled trials with repeated assessment of patient-reported outcomes: what correlation between baseline and follow-up outcomes should we assume?. Trials.

[38] Gore N, Bradshaw J, Hastings R (2022). Early positive approaches to support (E-PAtS): Qualitative experiences of a new support programme for family caregivers of young children with intellectual and developmental disabilities. J Appl Res Intellect Disabil.

[39] Coulman E, Hastings R, Gore N (2020). The Early Positive Approaches to Support (E-PAtS) study: study protocol for a feasibility cluster randomised controlled trial of a group programme (E-PAtS) for family caregivers of young children with intellectual disability. Pilot Feasibility Stud.

[40] Benjamini Y, Yekutieli D (2001). The control of the false discovery rate in multiple testing under dependency. Ann Statist.

[41] Dunn G, Maracy M, Tomenson B (2005). Estimating treatment effects from randomized clinical trials with noncompliance and loss to follow-up: the role of instrumental variable methods. Stat Methods Med Res.

[42] Moore GF, Audrey S, Barker M (2015). Process evaluation of complex interventions: Medical Research Council guidance. BMJ.

[43] Drummond MF, Sculpher MJ, Claxton K (2015). Methods for the economic evaluation of health care programmes.

[44] Claxton K, Martin S, Soares M (2015). Methods for the estimation of the National Institute for Health and Care Excellence cost-effectiveness threshold. Health Technol Assess.

[45] Faria R, Gomes M, Epstein D (2014). A guide to handling missing data in cost-effectiveness analysis conducted within randomised controlled trials. Pharmacoeconomics.

