# Supplementary material for: E-PAtS trial: a protocol for a cluster randomised controlled trial of Early Positive Approaches to Support (E-PAtS) for families of young children with intellectual disabilities
Source: BMJ Open. 2026 Oct 27;16(10):e112783. doi: 10.1136/bmjopen-2025-112783 (PMC13723794; doi:10.1136/bmjopen-2025-112783)
Supplement: Supplementary file 1 [file bmjopen-16-10-s001.pdf]

# PARTICIPANT CONSENT FORM

**Version V4.0_Approved_12.02.24**

## A Cluster Randomised Controlled Trial of Early Positive Approaches to Support (E-PAtS) for families of young children with intellectual disability.

**Name of researcher(s):** *Dr Nick Gore*

Please initial box

1. I have read and understood the information sheet (E-PAtS_Participant Information Sheet V4.0_Approved_12.02.24) for this study and I had the opportunity to ask questions about it.

2. I understand that my participation is voluntary and that I am free to withdraw at any time without giving any reason.

3. I understand that if I withdraw it may not be possible to destroy data about me (i.e., if the analysis has already been conducted or if reports/publications have been written)

4. I understand that information collected during the study may be looked at by members of the research team, and regulatory authorities supervising the study.

5. I understand that all information collected during the research study will be kept strictly confidential and processed in compliance with data protection legislation.

6. I understand that some of the study data will be processed by third parties (known as data processors), such as software and transcription companies, and that contractual terms, policies and procedures will be put in place to ensure confidentiality is respected.

7. I understand that any contact details that I share directly with the research team may be shared with the service providers (organisations who provide the E-PAtS programme).

8. I understand that if I am referred to the research team via the service provider, any further contact details that I share with the research team may be shared back with the service provider.



FUNDED BY NIHR | National Institute for Health and Care Research

9. I understand that I may or may not be selected to take part in an E-PAtS group straightaway and that this will be decided at random. ☐

10. I understand that if I am selected for the control group I will be offered an opportunity to attend E-PAtS at a later time (approximately 12 months later) ☐

11. I understand that I will be asked to complete questionnaires on three occasions, the last time being approximately one year after first agreeing to take part in the study. I understand that I am being asked to complete these questionnaires, whether or not I am taking part in an E-PAtS group straightaway. ☐

12. I consent to the study team contacting me, if the study does not have enough families to randomise, to ask me if I am able to make the date of a future E-PAtS programme. If I do not want this to happen, I will be withdrawn from the study. If I agree, I may be asked to repeat some of the eligibility checks and parts of the baseline questionnaire. ☐

13. I understand that I may be invited to take part in a short interview to answer questions about my experiences of taking part in the study and am happy to be invited. ☐

14. I understand that the information collected will be anonymized and used to support other research in the future. It may be shared anonymously with other researchers. ☐

15. I understand that the information collected for this study will be securely stored in line with University of Kent policies for up to 15 years. ☐

16. I understand confidentiality may need to be broken if the research team suspects that I or someone else is at risk of harm. ☐

17. I agree to take part in the above study. ☐



FUNDED BY NIHR | National Institute for Health and Care Research

| | | |
|---|---|---|
| Name of Participant | Date | Signature of participant |
| | | Signature of researcher (verbal consent) |
| Name of researcher taking consent | Date | Signature |

---

For Admin Purposes Only

Centre ID ____ Participant ID ____ Participant Initials ☐☐☐☐ Date form completed ☐☐ / ☐☐ / ☐☐☐☐

Day Month Year

This study is funded by the National Institute for Health and Care Research (NIHR) Public Health Research (PHR) Programme 21/550. The views expressed are those of the author(s) and not necessarily those of the NIHR or the Department of Health and Social Care
