# Supplementary material for: E-PAtS trial: a protocol for a cluster randomised controlled trial of Early Positive Approaches to Support (E-PAtS) for families of young children with intellectual disabilities
Source: BMJ Open. 2026 Oct 27;16(10):e112783. doi: 10.1136/bmjopen-2025-112783 (PMC13723794; doi:10.1136/bmjopen-2025-112783)
Supplement: Supplementary file 2 [file bmjopen-2025-112783.reviewer_comments.pdf]

PEER REVIEW HISTORY

BMJ Open publishes all reviews undertaken for accepted manuscripts. Reviewers are asked to complete a checklist review form and are provided with free text boxes to elaborate on their assessment. These free text comments are reproduced below.

ARTICLE DETAILS

Title (Provisional)

E-PAtS Trial: A Protocol for a Cluster Randomised Controlled Trial of Early Positive Approaches to Support (E-PAtS) for families of young children with intellectual disabilities

Authors

Williams-Thomas, Rhys; Coulman, Elinor; Bradshaw, Jill; Austin, Debbie; Bradshaw, Cameron; Gianatsi, Myrsini; Gillespie, David; Grant, Gemma; Hastings, Richard P.; Kim, Sungwook; May, Richard; McNamara, Rachel; Mulhall, Peter; Penrose, Lydia; Petrou, Stavros; Rose, Sophie; Segrott, Jeremy; Sweeney, Joanne; Taggart, Laurence; Tomlinson, Serena; Totsika, Vasiliki; Williams, Caitlin; Gore, Nick

---

VERSION 1 - REVIEW

**Reviewer**	**1**
**Name**	**Kelson, Zoe**
**Affiliation**	**University of Exeter, Mathematics**
**Date**	**30-Jun-2026**
**COI**	**None**

This protocol describes a randomised controlled trial of the E-PAtS intervention, a programme tailored for families of young children with intellectual disabilities.

Reviewer comments:

"This protocol received approval from Humanities and Social Sciences Ethics Committee, University of Birmingham (13/08/2025, Ref: ERN_3848-Jul2025), following initial approval from the Tizard Centre University of Kent Ethics Committee (16/08/2023, Ref: 0897)"

Can the authors please confirm the trial has been registered and provide the study registration details?

"The protocol has been written according to the Standard Protocol Items: Recommendations for Interventional Trials (SPIRIT) statement, the intervention according to the template for intervention description and replication (TIDieR) checklist, and the final report will follow the Consolidated Standards of Reporting Trials (CONSORT) statement. "

Thanks to the authors for providing a copy of the SPIRIT checklist, and confirming adherence to reporting guidelines.

"The recruitment start date and the approximate recruitment end date are 01/09/2023 and 31/01/2026 respectively"

Can these study dates please also be specified in the Abstract?

Can the proposed statistical methods please be briefly summarised in the Abstract by the authors?

"The primary objective is to assess whether the E-PAtS intervention has a positive effect on primary parents/carers' mental wellbeing at the 12 month time point, assessed by Warwick-Edinburgh Mental Well-Being Scale. Secondary objectives include further exploration of mental health outcomes in both parent and child as well as health related quality of life and an economic analysis." [Abstract]

and

"The primary outcome measure will be the Warwick-Edinburgh Mental Well-Being Scale (WEMWBS) to measure parental/carers mental wellbeing (24) at 12 months post-randomisation...

Secondary outcome measures include: 1. perceptions of parenting: Me as a Parent Scale short form (25); 2. parental/carer anxiety and depression: Hospital Anxiety and Depression scale (26); 3. child with intellectual disability adaptive development: The GO4KIDDS Brief Adaptive Scale (27) adapted to include an additional communication item (completed by parents/carers of children 18 months and above); 4. child emotional and behavioural development: The Strengths and Difficulties Questionnaire (completed by parents/carers of children 2 years and above) (28); 5. child sleep: Single Sleep question from Millennium Cohort Survey (29); 6. child health-related quality of life: The Pediatric Quality of Life Inventory TM Version 4.0 Generic Core Scales (completed by parents/carers of children 2 years and above); 7. family functioning: Family APGAR scale (30); 8. support for family: Family Support Scale (31); 9. Parent health-related quality of life (EQ-5D-5L (32); 10. Client Service Receipt Inventory (CSRI) modified from the feasibility study (23)"

The study outcomes are clearly defined by the authors in the protocol.

Can the authors please pre-specify any subgroup analyses they plan on exploring in the protocol?

"Participants will be recruited across in England, Scotland and Northern Ireland across 4 research centres. Third sector and local authority funded service provider organisations will be selected to take part in the trial if they are able to deliver E-PAtS with a minimum group number of 6 families and are willing to refer potentially eligible families to the research team"

Can the authors please clarify how they will assess whether the included cohort is representative?

"Participants for this study will be adult family carers of children with intellectual disabilities. At least one family carer (first family carer) is recruited from each family, with a second family carer then recruited where possible. First family carers attend the E-PAtS intervention if randomised to do so. Second family carers are able to attend all, some or none of the E-PAtS sessions. "

Can the authors please clarify whether family is the cluster in this study here?

Can the authors please comment on whether differences by centre and country will be explored?

"A total of 458 families (229 per arm) will be randomised in this study, to include a total of 253 approximately 594 family carers (297 per arm). The sample size is estimated for 90% power, with a 254 two-sided Type I error rate of 5%. The established minimally important difference on the selected 255 primary outcome measure, the Warwick and Edinburgh Mental Well-Being Scale (WEMWBS) scale, is 256 3 points (24, 35), assuming a standard deviation of 11 points (i.e., a standardised effect size of 0.27) 257 and a correlation of 0.5 between family carers' baseline and follow-up WEMWBS scores. This is lower 258 than the correlation observed in the feasibility study (0.57) and is in line with baseline to follow-up 259 correlations reported elsewhere (36, 37). Family Carer scores are expected to cluster within families 260 (intra-class correlation of 0.4 assuming average of 1.3 participants per family), but families are not 261 expected to cluster within intervention groups. These assumptions were informed by the results of 262 our feasibility trial (38, 39), in which the intra-class correlation of responses within families was less 263 than 0.3, with no evidence of clustering by intervention group. A 20% loss to follow-up is allowed, 264 which is lower than we found in our feasibility study but is expected given the enhanced retention 265 strategy to be used."

The authors have suitably communicated their assessment of sample size.

"Any interim analyses requiring knowledge of treatment allocation will be performed by a study-independent statistician. "

Can the authors please clarify how this interim analysis impacts on study power, and adjust the sample size assessment accordingly?

"The First Family Carer will complete informed consent, either online via an email link to the online consent or verbally with a researcher over the telephone or on video conferencing, as per participant preference. Baseline data will be collected in the same way, as per participant preference...

Participant questionnaire data for this study are collected at baseline, 4-months post-randomisation and 12-months post-randomisation, collected either remotely with the

support of a researcher or via online link as per participant preference. See Table 1 for details of all outcome measures and data collection timepoints. "

and

"Data validation will be performed at entry to reduce missing or unexpected data."

Can the authors please confirm how they will assess missing data to determine whether it can be assumed to be missing at random?

Furthermore, can the authors please specify how they plan on handling missing data in the analysis?

" The following strategies will be utilised to maximise retention: .."

The authors clearly describe how they plan to minimise attrition.

"After completing baseline assessments, families are randomised on a 1:1 basis either to the E-PAtS intervention or continue with usual care... " [Abstract]

and

"The units of randomisation are families including one or two family carer participants. Cluster randomisation was chosen to avoid contamination between family members, and to reflect intended implementation at the family level. Following consent, families will be randomised to E-PAtS and UP or UP alone in groups of 6 to 16. A group of families will be randomised simultaneously using a single permuted block of the same size as the group, ensuring that allocations within the group are balanced between the intervention and control arms, with 3 to 8 families in the intervention arm. "

Can the authors please confirm if there are any stratification variables in the randomisation?

"Data will be summarised using frequencies with percentages, means with standard deviations, and medians with interquartile ranges as appropriate"

Can the authors please specify how they plan on assessing normality?

"Differences in WEMWBS (24,35) scores will be estimated at follow-up using multilevel regression to account for clustering of participants within families"

and

"Secondary outcomes will be analysed similarly. We will correct for multiple hypothesis testing by controlling the false discovery rate (FDR) (40)."

Appropriate modelling methods are described, suitably adjusting for clustering in the analytical design.

"The analysis population will be defined using the intention-to-treat (ITT) approach"

Can the authors please clarify whether they will also explore a per protocol (PP) sensitivity analysis?

"In the analysis, the effect of being offered E-PAtS (ITT) will be investigated. To also investigate the effect of E-PAtS receipt, a complier average causal effect (CACE) analysis will be used...

For child outcomes, an additional sensitivity analysis will be conducted in which only the first carers' responses will be considered"

Insightful additional analyses are specified by the authors.

"cost-utility analysis will be conducted and expressed in terms of incremental cost per QALY gained. Bivariate regression of costs and QALYs, with multiple imputation of missing data, will be conducted to generate within-trial estimates of incremental cost-effectiveness associated with E-PAtS. Sensitivity analyses will assess the impact of areas of uncertainty surrounding aspects of the economic evaluation. Cost-effectiveness acceptability curves will be used to show the probability of cost-effectiveness of E-PAtS at alternative threshold values for an additional QALY held by decision-makers (44). A separate cost-consequences analysis will be conducted that presents the disparate costs and consequences associated with E-PAtS in disaggregated form. "

A valuable economic evaluation is planned.

Can the authors please provide more detail on the planned multiple imputation for missing data?

"Some settings in which E-PAtS is delivered (i.e., health services) are not in scope for this trial
• Participants for this trial will not be blind to their allocation " [Strengths and Limitations]

Can the study limitations please be further addressed in the Discussion?

VERSION 1 - AUTHOR RESPONSE

Reviewer: 1
Prof. Zoe Kelson, University of Exeter
Comments to the Author:
This protocol describes a randomised controlled trial of the E-PAtS intervention, a programme tailored for families of young children with intellectual disabilities.

Reviewer comments:

Reviewer Comment 1:
"This protocol received approval from Humanities and Social Sciences Ethics Committee, University of Birmingham (13/08/2025, Ref: ERN_3848-Jul2025), following initial approval from the Tizard Centre University of Kent Ethics Committee (16/08/2023, Ref: 0897)"
Can the authors please confirm the trial has been registered and provide the study registration details?

Response:
The trial has been registered and has the ISRCTN registration number 11192967, this was included under trial registration, line 60 and 61 of the manuscript. Additionally, details have

been added to the study status section.

Reviewer Comment 2:
"The protocol has been written according to the Standard Protocol Items: Recommendations for Interventional Trials (SPIRIT) statement, the intervention according to the template for intervention description and replication (TIDieR) checklist, and the final report will follow the Consolidated Standards of Reporting Trials (CONSORT) statement. "
Thanks to the authors for providing a copy of the SPIRIT checklist and confirming adherence to reporting guidelines.

Response:
Thank you, SPIRIT checklist has been updated in-line with editor comments to include "not applicable" for sections that aren't used as part of this study design where previously there were some blank fields.

Reviewer Comment 3:
"The recruitment start date and the approximate recruitment end date are 01/09/2023 and 31/01/2026 respectively"
Can these study dates please also be specified in the Abstract?

Can the proposed statistical methods please be briefly summarised in the Abstract by the authors?

Response:
Yes, the study dates have been added to the revised abstract as well as briefly summarising the methods:

"A recruitment period of 29 months will commence on 1 September 2023."

"Mental Well-Being Scale. The primary treatment effect at 12 months will be estimated using a partially clustered linear mixed model of repeated WEMWBS measurements, adjusting for baseline score and site, with random effects for participants, families, and intervention-arm E-PAtS groups. Secondary objectives"

Reviewer Comment 4:
"The primary objective is to assess whether the E-PAtS intervention has a positive effect on primary parents/carers' mental wellbeing at the 12-month time point, assessed by Warwick-Edinburgh Mental Well-Being Scale. Secondary objectives include further exploration of mental health outcomes in both parent and child as well as health related quality of life and an economic analysis." [Abstract]
and
"The primary outcome measure will be the Warwick-Edinburgh Mental Well-Being Scale (WEMWBS) to measure parental/carers mental wellbeing (24) at 12 months post-randomisation…
Secondary outcome measures include: 1. perceptions of parenting: Me as a Parent Scale short form (25); 2. parental/carer anxiety and depression: Hospital Anxiety and Depression scale (26); 3. child with intellectual disability adaptive development: The GO4KIDDS Brief Adaptive Scale (27) adapted to include an additional communication item (completed by parents/carers of children 18 months and above); 4. child emotional and behavioural development: The Strengths and Difficulties Questionnaire (completed by parents/carers of children 2 years and above) (28); 5. child sleep: Single Sleep question from Millennium Cohort Survey (29); 6. child health-related

quality of life: The Pediatric Quality of Life Inventory TM Version 4.0 Generic Core Scales (completed by parents/carers of children 2 years and above); 7. family functioning: Family APGAR scale (30); 8. support for family: Family Support Scale (31); 9. Parent health-related quality of life (EQ-5D-5L (32); 10. Client Service Receipt Inventory (CSRI) modified from the feasibility study (23)"
The study outcomes are clearly defined by the authors in the protocol.

Response:
Thank you for the comment, we're glad that the outcomes are clearly defined.

Reviewer Comment 5:
Can the authors please pre-specify any subgroup analyses they plan on exploring in the protocol?

Response:
We will explore whether the effect of the E-PAtS programme on WEMWBS scores differs between online and in-person delivery as a subgroup analysis. Details have now been provided at the "Main analysis" section:

"The potential for differential effects of the E-PAtS programme on WEMWBS scores by intended mode of delivery (online vs. in-person) will be explored."

Reviewer Comment 6:
"Participants will be recruited from England, Scotland, Wales and Northern Ireland across 4 research centres. Third sector and local authority funded service provider organisations will be selected to take part in the trial if they are able to deliver E-PAtS with a minimum group number of 6 families and are willing to refer potentially eligible families to the research team"
Can the authors please clarify how they will assess whether the included cohort is representative?

Response:
Although we will not be able to establish if the recruited sample is representative of the population of families of young children with learning disability, we will compare key demographic characteristics (e.g., ethnicity) of the sample with UK population norms. Families will be recruited from a range of geographic areas across the UK and from varied environments (e.g., both rural and inner-city areas).

Reviewer Comment 7:
"Participants for this study will be adult family carers of children with intellectual disabilities. At least one family carer (first family carer) is recruited from each family, with a second family carer then recruited where possible. First family carers attend the E-PAtS intervention if randomised to do so. Second family carers are able to attend all, some or none of the E-PAtS sessions. "
Can the authors please clarify whether family is the cluster in this study here?

Response:
Correct, the family is the cluster, the units of randomisation are families including one or two family carer participants. Cluster-randomisation was chosen to avoid contamination between family members, and to reflect intended implementation at the family level. Further text has been added in the manuscript to include this detail in line with reviewer comment 13 which

provides further detail on stratification variables.

Reviewer Comment 8:
Can the authors please comment on whether differences by centre and country will be explored?

Response:
Study site is included in the analysis, the below text has been added to the abstract and the 2nd paragraph of text added to the randomisation section:
"Mental Well-Being Scale. The primary treatment effect at 12 months will be estimated using a partially clustered linear mixed model of repeated WEMWBS measurements, adjusting for baseline score and site, with random effects for participants, families, and intervention-arm E-PAtS groups. Secondary objectives"

Following consent, families will be randomised 1:1 to E-PAtS and UP or UP and waitlist control, stratified by site. Families will be randomised in batches of 6 to 16 two-weeks before the planned E-PAtS program starts at each service provider, using randomly permuted blocks of varying size to reduce imbalance and maintain allocation concealment.

Reviewer Comment 9:
"A total of 458 families (229 per arm) will be randomised in this study, to include a total of 253 approximately 594 family carers (297 per arm). The sample size is estimated for 90% power, with a 254 two-sided Type I error rate of 5%. The established minimally important difference on the selected 255 primary outcome measure, the Warwick and Edinburgh Mental Well-Being Scale (WEMWBS) scale, is 256 3 points (24, 35), assuming a standard deviation of 11 points (i.e., a standardised effect size of 0.27) 257 and a correlation of 0.5 between family carers' baseline and follow-up WEMWBS scores. This is lower 258 than the correlation observed in the feasibility study (0.57) and is in line with baseline to follow-up 259 correlations reported elsewhere (36, 37). Family Carer scores are expected to cluster within families 260 (intra-class correlation of 0.4 assuming average of 1.3 participants per family), but families are not 261 expected to cluster within intervention groups. These assumptions were informed by the results of 262 our feasibility trial (38, 39), in which the intra-class correlation of responses within families was less 263 than 0.3, with no evidence of clustering by intervention group. A 20% loss to follow-up is allowed, 264 which is lower than we found in our feasibility study but is expected given the enhanced retention 265 strategy to be used."
The authors have suitably communicated their assessment of sample size.

Response:
Thank you for the positive comment.

Reviewer Comment 10:
"Any interim analyses requiring knowledge of treatment allocation will be performed by a study-independent statistician. "
Can the authors please clarify how this interim analysis impacts on study power, and adjust the sample size assessment accordingly?

Response:
No interim analysis was planned for the study, the sample size was recalculated based on the internal pilot criteria (recruitment, retention and adherence).

Reviewer Comment 11:
"The First Family Carer will complete informed consent, either online via an email link to the online consent or verbally with a researcher over the telephone or on video conferencing, as per participant preference. Baseline data will be collected in the same way, as per participant preference...
Participant questionnaire data for this study are collected at baseline, 4-months post-randomisation and 12-months post-randomisation, collected either remotely with the support of a researcher or via online link as per participant preference. See Table 1 for details of all outcome measures and data collection timepoints. "
and
"Data validation will be performed at entry to reduce missing or unexpected data."
Can the authors please confirm how they will assess missing data to determine whether it can be assumed to be missing at random?
Furthermore, can the authors please specify how they plan on handling missing data in the analysis?

Response:
Thank you for your comment, the revised manuscript has been edited to include more detail on how missing data will be defined and handled during the analysis:

"Missing outcome data will be summarised by trial arm and timepoint, including recorded reasons for missingness where available. The partially clustered linear mixed model will use all available outcome data and provide estimates under a missing-at-random assumption."

Reviewer Comment 12:
" The following strategies will be utilised to maximise retention: .."
The authors clearly describe how they plan to minimise attrition.

Response:
Thank you for the positive comment.

Reviewer Comment 13:
"After completing baseline assessments, families are randomised on a 1:1 basis either to the E-PAtS intervention or continue with usual care... " [Abstract]
and
"The units of randomisation are families including one or two family carer participants. Cluster randomisation was chosen to avoid contamination between family members, and to reflect intended implementation at the family level. Following consent, families will be randomised to E-PAtS and UP or UP alone in groups of 6 to 16. A group of families will be randomised simultaneously using a single permuted block of the same size as the group, ensuring that allocations within the group are balanced between the intervention and control arms, with 3 to 8 families in the intervention arm. "
Can the authors please confirm if there are any stratification variables in the randomisation?

Response:
Thank you for the comment, there are stratification variables for the randomisation and the revised manuscript has been edited to include further detail:

"The units of randomisation are families including one or two family carer participants. Cluster-randomisation was chosen to avoid contamination between family members, and to reflect

intended implementation at the family level. Following consent, families will be randomised 1:1 to E-PAtS and UP or UP and waitlist control, stratified by site. Families will be randomised in batches of 6 to 16 two-weeks before the planned E-PAtS program starts at each service provider, using randomly permuted blocks of varying size to reduce imbalance and maintain allocation concealment."

Reviewer Comment 14:
"Data will be summarised using frequencies with percentages, means with standard deviations, and medians with interquartile ranges as appropriate"
Can the authors please specify how they plan on assessing normality?

Response:

Thank you for the comment, the manuscript has been updated to specify how we're assessing normality:

"Model assumptions will be assessed using diagnostic plots. Normality will be assessed using quantile–quantile plots of the model residuals, while residual-versus-fitted plots will be examined to assess linearity and homoscedasticity."

Reviewer Comment 15:
"Differences in WEMWBS (24,35) scores will be estimated at follow-up using multilevel regression to account for clustering of participants within families"
and
"Secondary outcomes will be analysed similarly. We will correct for multiple hypothesis testing by controlling the false discovery rate (FDR) (40)."
Appropriate modelling methods are described, suitably adjusting for clustering in the analytical design.

Response:
Thank you for the positive comment.

Reviewer Comment 16:
"The analysis population will be defined using the intention-to-treat (ITT) approach"
Can the authors please clarify whether they will also explore a per protocol (PP) sensitivity analysis?

Response:
There is no per-protocol sensitivity analysis planned. Instead, the primary analysis will follow the intention-to-treat principle, and the effect among families who would comply with the intervention if offered it will be explored using a complier average causal effect (CACE) analysis.

Reviewer Comment 17:
"In the analysis, the effect of being offered E-PAtS (ITT) will be investigated. To also investigate the effect of E-PAtS receipt, a complier average causal effect (CACE) analysis will be used... For child outcomes, an additional sensitivity analysis will be conducted in which only the first carers' responses will be considered"
Insightful additional analyses are specified by the authors.

Response:

Thank you for the positive comment.

Reviewer Comment 18:
"cost-utility analysis will be conducted and expressed in terms of incremental cost per QALY gained. Bivariate regression of costs and QALYs, with multiple imputation of missing data, will be conducted to generate within-trial estimates of incremental cost-effectiveness associated with E-PAtS. Sensitivity analyses will assess the impact of areas of uncertainty surrounding aspects of the economic evaluation. Cost-effectiveness acceptability curves will be used to show the probability of cost-effectiveness of E-PAtS at alternative threshold values for an additional QALY held by decision-makers (44). A separate cost-consequences analysis will be conducted that presents the disparate costs and consequences associated with E-PAtS in disaggregated form. "
A valuable economic evaluation is planned.
Can the authors please provide more detail on the planned multiple imputation for missing data?

Response:
Thank you for your comment. Before analysing the trial data, we will check for missing values and determine their causes using the Faria et al.'s framework [1]. We will analyse the nature of this missing data to see if it qualifies as Missing at Random (MAR). If it is MAR, we will apply multiple imputation methods.

Reviewer Comment 19:
"Some settings in which E-PAtS is delivered (i.e., health services) are not in scope for this trial • Participants for this trial will not be blind to their allocation " [Strengths and Limitations]
Can the study limitations please be further addressed in the Discussion?

Response:
Thank you for the comment, the discussion section has been updated to include some further detail on the strengths and weaknesses:

'The current RCT of E-PAtS has the following notable strengths:

1. Grounded in long term and substantive PPIE input: The E-PAtS intervention has been co-produced with family carers and is co-facilitated by family carers and partners working in partnership; PPIE co-applicants and partners supported development of the study design and strategies for recruitment and retention and a PPIE group will advise on processes, materials and dissemination activities throughout the study.
2. Study design (a two-arm cluster randomised wait-list control trial, with multi-point recruitment across the UK) is a robust design that builds on findings from prior feasibility testing and analysis employs appropriate modelling methods that suitably adjust for clustering and include carefully considered sensitivity analysis.
3. Inclusion of embedded process evaluation and health economics analysis.

The study has the following limitations:

1. Health services are not in scope for this study. This was a pragmatic decision (based on available resource and timelines) to focus on a single setting that is the most common setting for delivery in practice. We have no reason to suggest the intervention itself would work differently in health settings.

2. It was not possible to blind participants to allocation due to the nature of the intervention. However, outcome assessors will be blind to allocation'

References

1. Faria, R., Gomes, M., Epstein, D., and White, I.R., *A guide to handling missing data in cost-effectiveness analysis conducted within randomised controlled trials*. Pharmacoeconomics, 2014. **32**(12): p. 1157-1170.

VERSION 2 - REVIEW

**Reviewer**	**1**
**Name**	**Kelson, Zoe**
**Affiliation**	**University of Exeter, Mathematics**
**Date**	**09-Sep-2026**
**COI**	

Many thanks to the authors for their responses and revisions to the protocol.

Can the following responses to reviewer please be expanded on within the article by the authors, including providing more detail regarding the planned multiple imputation methods (e.g. what model, and how many iterations)?

"Although we will not be able to establish if the recruited sample is representative of the population of families of young children with learning disability, we will compare key demographic characteristics (e.g., ethnicity) of the sample with UK population norms. Families will be recruited from a range of geographic areas across the UK and from varied environments (e.g., both rural and inner-city areas). "

and

"Before analysing the trial data, we will check for missing values and determine their causes using the Faria et al.'s framework [1]. We will analyse the nature of this missing data to see if it qualifies as Missing at Random (MAR). If it is MAR, we will apply multiple imputation methods."

VERSION 2 - AUTHOR RESPONSE

Reviewer Comment 1:

Reviewer: 1
Prof. Zoe Kelson, University of Exeter
Comments to the Author:
Many thanks to the authors for their responses and revisions to the protocol.

Can the following responses to reviewer please be expanded on within the article by the authors, including providing more detail regarding the planned multiple imputation methods (e.g. what model, and how many iterations)?
"Although we will not be able to establish if the recruited sample is representative of the population of families of young children with learning disability, we will compare key demographic characteristics (e.g., ethnicity) of the sample with UK population norms. Families will be recruited from a range of geographic areas across the UK and from varied environments (e.g., both rural and inner-city areas). "
and
"Before analysing the trial data, we will check for missing values and determine their causes using the Faria et al.'s framework [1]. We will analyse the nature of this missing data to see if it qualifies as Missing at Random (MAR). If it is MAR, we will apply multiple imputation methods."

Response:
We have amended the main document to reflect these changes:

"Baseline demographic characteristics will be summarised descriptively. Where appropriate, key demographic characteristics of the recruited sample, such as ethnicity, will also be descriptively compared with relevant UK population data. These comparisons will not be used to formally assess the representativeness of the trial sample."

"Before analysing the trial data, we will check for missing values and determine their causes using the Faria et al.'s framework [45]. We will analyse the nature of this missing data to see if it qualifies as Missing at Random (MAR). If it is MAR, we will apply multiple imputation methods."

I'm currently out of the office for some time, if possible can any further correspondence about the publication of this paper be sent to Elinor Coulman.
